# Oculogyric crisis symptoms related to risperidone treatment: a case report

**DOI:** 10.1186/s12888-023-05379-3

**Published:** 2023-11-24

**Authors:** Tao Lv, Liping Wu, Longlong Li, Min Zhang, Qingyu Tan, Ping Liu

**Affiliations:** The People’s Hospital of Deyang, 173 Section One, North Taishan Road, Deyang, Sichuan 618000 China

**Keywords:** Risperidone, Clozapine, Oculogyric crisis, Therapeutic drug monitoring

## Abstract

**Background:**

Oculogyric crisis (OGC) is a rare focal dystonia of the ocular muscles that not only interferes with patients’ medication adherence but also negatively affects the course and prognosis of the primary disease. Early detection and treatment of OGC can improve patients’ medication adherence and quality of life.

**Case presentation:**

This paper reports a case of a 19-year-old Asian female with a diagnosis of schizophrenia who was treated intermittently with atypical antipsychotics aripiprazole or risperidone for 2 years, with improvement of psychotic symptoms during the course of medication, and then developed double eye rolling and staring with irritability when treated with risperidone 4 mg/d or 6 mg/d. Then, we changed the medication to clozapine, and the patient’s psychotic symptoms were controlled and stable. The symptoms of double eye rolling and gaze disappeared.

**Conclusion:**

Oculogyric crisis (OGC) is a rare focal dystonia of the oculogyric muscle. This case provides clinicians with a basis for the early recognition and management of oculogyric crisis during the use of atypical antipsychotics (risperidone).

Oculogyric crisis (OGC) is a rare focal dystonia of the oculogyric muscle, characterized by episodes of bilateral involuntary ocular jerking with fixed upwards gaze in both eyes lasting minutes to hours, usually accompanied by agitation, and occurring in acute and chronic episodes [1]. It is commonly seen in young males using typical antipsychotics with high effectiveness, antipsychotics with high doses, and parenteral administration [2]. The exact prevalence of OGC is unknown, although in one study, it was reported to be approximately 5.3% [3], while the prevalence of OGC with antipsychotic medication was reported to range from 0.9% to 3.4% [4].

In this paper, we report the presentation and management of a case of a young Asian woman who developed an oculogyric crisis while using a therapeutic dose of risperidone to provide a reference point for clinicians when using risperidone, especially not to ignore the presentation of side effects in female patients when using risperidone.

## Case history

**Fig. 1:**
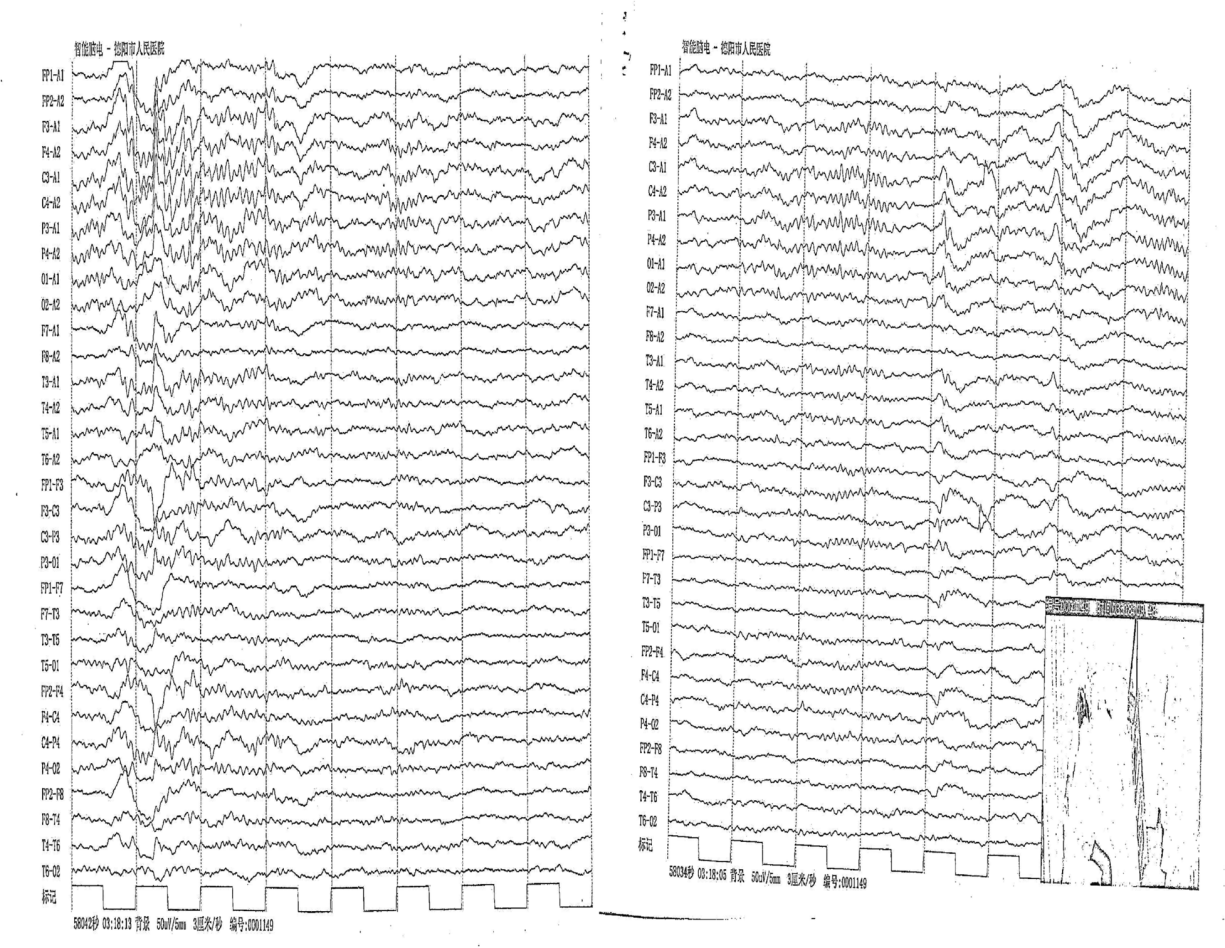
EEG

**Fig. 2:**
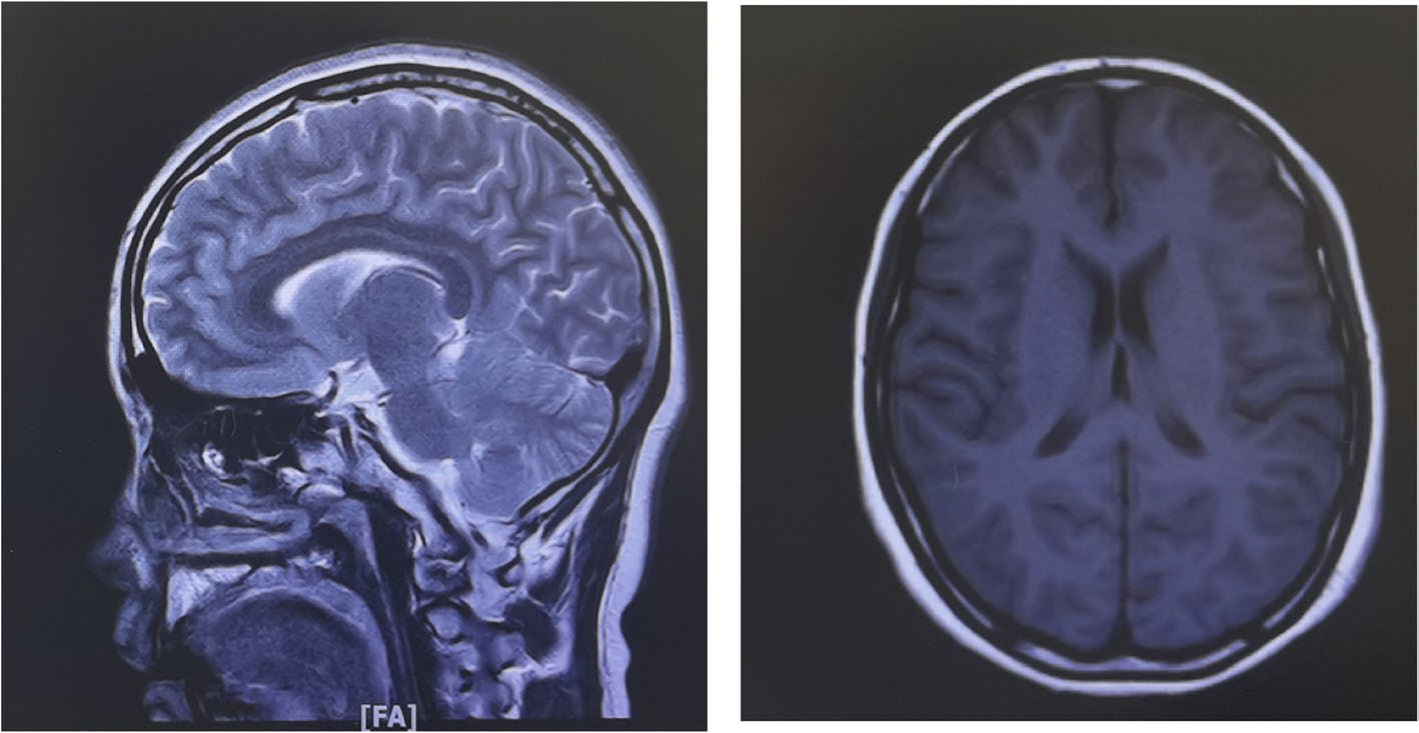
MRI

A 19-year-old Asian woman with schizophrenia was treated intermittently with aripiprazole and risperidone for the past 2 years, with stable control of psychotic symptoms during the medication period. She discontinued the medication on her own after the first treatment when her family perceived symptom relief. The second hospital admission, the patient appeared to raise her eyes, stare, and attacks associated with irritability again, we considered it was an extravertebral collateral. When using trihexyphenidyl hydrochloride (( +) α-Cyclohexyl-α-phenyl-1-piperidinepropanol hydrochloride) 4 mg/d, her symptoms were partially relieved, but it is not completely relieved and there is still a recurrence. Her psychotic symptoms were relieved by medication, and she was able to study as usual. However, she refused to take the medication because of recurrent episodes of double eye rolling and staring, preceded by irritability and lasting from a few minutes to half an hour each time, and she did not experience double eye rolling and staring after discontinuing the medication. The third hospital admission, considering that the patient was a young female, poor medication compliance, and family communication, suggested switching to a long-acting injection (paliperidone palmitate injection) to improve treatment compliance, risperidone alone during the hospital admission, and increasing risperidone to 4 mg—6 mg/day. The patient appeared to raise her eyes, stare, and attacks associated with irritability again, and each attack lasted a few minutes to approximately 30 min, we considered it was an extravertebral collateral. When using trihexyphenidyl hydrochloride (( +) α-Cyclohexyl-α-phenyl-1-piperidinepropanol hydrochloride) 4 mg/d, her symptoms were partially relieved, but it is not completely relieved and there is still a recurrence. We performed a video EEG (see Fig. 1), MRI (see Fig. 2), and cerebrospinal fluid examination (see Table 1), all of examination were normal, and we considered the patient's symptoms to be OGC. Then risperidone was gradually discontinued, and the patient was switched to clozapine at a dose of 125 mg/day. Her psychotic symptoms were controlled and stabilized. She did not suffer from double eye rolling and staring symptoms. The patient signed an informed consent form for publication. See Table 2.

**Table 1:** Cerebrospinal fluid examination

Items	Results
NMDA IgG	negative
AMPA1 IgG	negative
AMPA2 IgG	negative
LGI IgG	negative
GABAB IgG	negative
CASPR2 IgG	negative

**Table 2:** Changes in patient history and drug use

Treat environment	Time	Symptoms	Complementary check	Diagnosis	Main Medication	With or without OGC
Hospitalization (First time)	April 15, 2021-September 2021	Verbal Hallucinations, inhibition of thought, delusion of reference, inattention, anxiety, no self-awareness	CSF routine, biochemistry, and autoimmune encephalitis were unremarkable, and video EEG and MRI of the cranial brain were normal	Acute schizophrenia-like psychotic disorder	Aripiprazole 30 mg/d Risperidone 2 mg/d	without
outpatient service	September 2021-March 2022	Positive symptoms in remission, no self-awareness			Self-discontinuation of medication	without
Hospitalization (Second time)	March 17,2022- August 2022	Verbal hallucinations, delusions of victimization, feelings of being watched and controlled, delusions of physical influence, no self-awareness	video EEG and MRI of the cranial brain were normal	schizophrenia	Aripiprazole 20 mg/d Risperidone 6 mg/d	With OGC(Episodes of upwards rolling eyes, gazing, closed eyes are seen on both sides of the upper eyelid muscles, involuntary trembling, cannot control themselves, feeling pain and irritability, seizure process is clear consciousness, no limbic spasms, urinary and faeces incontinence and other manifestations, lasting a few minutes to half an hour, and self-relieving)
outpatient service	September 2022-April 2023				Intermittent medication	
Hospitalization (Third time)	April 13, 2023—April 15, 2023	Verbal Hallucinations, inhibition of thought, no self-awareness			Risperidone 6 mg/d	
	April 16, 2023-April 19, 2023				Risperidone 4 mg/d	
hospitalization + outpatient service	April 20, 2023, to present	Symptoms in remission, she was able to go to school			clozapine 125 mg/d	without

## Discussion

The oculogyric crisis is an acute dystonic reaction first described in patients with Parkinson’s disease in 1930 [5], characterized by persistent, bilateral, upwards deviation of the eyes, and it is a relatively rare extrapyramidal side effect of antipsychotic medication [6]. The pathophysiology of OGC is thought to be due to blockade of dopaminergic neurotransmitters in the substantia nigra striata pathway affecting the extraocular muscles [7]. Most commonly seen with typical antipsychotics and less commonly with atypical antipsychotics, oral anticholinergic medications can improve symptoms, but sometimes it is necessary to reduce or discontinue antipsychotic medication or switch to safer alternatives such as clozapine [8]. Although OGC is not fatal, it can cause severe pain and discomfort (increased patient fatigue and sleep disturbance) and an increased risk for nonadherence [9], social avoidance due to increased stigma, and ultimately serious psychological and physical effects on the patient [10]. As in the present case, the patient refused to take the medication due to an oculogyric crisis and had poor adherence.

Risperidone belongs to the group of atypical antipsychotics with dopamine and 5-hydroxyptamine receptor antagonism, which have a high affinity for 5-HT2A and DA2 receptors and exert their therapeutic effects in schizophrenia through dopamine antagonism [11]. It is usually independent of OGC, especially when used at low doses [8]. The use of risperidone at a dosage of 2 mg/day in the present case did not lead to OGC symptoms. The patient developed double eye rolling and staring when risperidone was increased to 4 mg/day or 6 mg/day. This effect was alleviated when we added trihexyphenidyl hydrochloride (( +) α-Cyclohexyl-α-phenyl-1-piperidinepropanol hydrochloride) [12]. When the risperidone dose was gradually reduced and replaced by clozapine, the symptoms did not recur, and the control of psychiatric symptoms was stabilized. It is suggested that the presence of oculogyric crisis in this case may have had no correlation with aripiprazole and may have been related to risperidone or even to risperidone dose. The occurrence of OGC is related to the type of antipsychotic drug on the one hand. Aripiprazole, as a partial antagonist of the limbic pathway of the midbrain, and as a partial agonist of D2, exerts a limited effect on dopamine in the nigrastriatal pathway, with a low incidence of extrapyramidal reactions [13]. In contrast, risperidone has a high affinity for both 5-HT2A and DA2 receptors, and has a high probability of acute extravertebral reactions and tardive dyskinesia. On the other hand, the occurrence of OGC may be related to the starting dose of antipsychotic drugs or the rate of dose increase. The plasma concentration of antipsychotic drugs is closely related to the occupancy of dopamine D2 receptors, and when the plasma drug is gradually increased to effective measurement, the plasma drug concentration and dopamine D2 receptor occupancy increase [14]. However, in the case of male schizophrenia, it was found that oculogyric crisis can be induced by the use of low doses of risperidone [15]. However, the patient in this case was a woman with schizophrenia and did not develop OGC at low doses of risperidone, and gender may be a risk factor for OGC at low doses of risperidone. Another study reported that age, gender, intellectual disability, and comorbid Tourette syndrome may be contributing factors to these risk factors that contribute to OGC at low doses of risperidone [16].

Clozapine is an atypical antipsychotic with a low risk of causing delayed dyskinesia [17], and it is often considered an effective treatment for patients with extrapyramidal reactions [18]. This advantage is related to the atypical biochemical profile of clozapine, specifically associated with high dopamine D_1_ receptor blockade and low dopamine D_2_ receptor blockade, which prevents an imbalance in dopamine D_1_/D_2_ receptor function to the extent that it does not induce extrapyramidal side effects [19]. However, a case report on the medication of a 14-year-old female patient with schizophrenia found that during treatment with clozapine, the patient’s psychotic symptoms improved, but adverse effects such as salivation, oculogyric crisis, slurred speech, and dysphagia occurred [20]. However, in this case, we found that after the patient presented oculogyric crisis, we gradually reduced the risperidone and switched to clozapine, the patient’s double eye rolling and staring disappeared, and her psychiatric symptoms were stabilized.

The Neuropsychopharmacology Consensus Guidelines for Therapeutic Drug Testing [21] have listed atypical antipsychotics such as amisulpride, clozapine, and olanzapine in the highest recommended category of therapeutic drug monitoring (TDM), while other atypical antipsychotics are rarely subjected to TDM. This case shows that the patient had a rare adverse reaction after taking atypical antipsychotics, which also suggests the need for TDM testing for atypical antipsychotics. In clinical practice, despite the challenging nature of recognizing and accurately diagnosing OGC, OGC not only affects adherence to medication but also has a negative impact on the course and prognosis of the primary disease; therefore, early detection and treatment play an important role in the management of OGC, which can improve adherence and the patient’s quality of life. In this case, the patient had recurrent symptoms of double eye rolling and staring, and the patient’s symptoms were not recognized as an oculogyric crisis but were simply considered an extrapyramidal side effect, with dose reduction of the medication or coadministration of anticholinergic medication. Although the patient’s symptoms were slightly improved, there was no complete resolution of the symptoms. After the second hospitalization, the patient had recurrent symptoms of double eye rolling and staring, and the patient felt that the symptoms were painful and was unwilling to take medication, which reduced treatment compliance. Therefore, it is crucial to recognize the oculogyric crisis at an early stage in clinical practice. Due to the combination of aripiprazole and risperidone in this case, drug interactions cannot be completely ruled out. It remains to be investigated whether the use of aripiprazole in combination with risperidone is more likely to lead to the occurrence of akinetic eye crisis.

## Conclusion

In conclusion, this case report of a risperidone-induced oculogyric crisis allows clinicians to recognize and routinely manage OGC for better clinical management and treatment.

## Data Availability

Not applicable.

## References

[1] Mahal P, Suthar N (2021). Spotlight on oculogyric crisis: a review. Indian J Psychol Med.

[2] Divac N, Prostran M, Jakovcevski I, et al. Second-generation antipsychotics and extrapyramidal adverse effects. Biomed Res Int. 2014;2014:656370. 10.1155/2014/656370 PMC4065707 24995318

[3] Lewis K, O’Day CS (2019). Dystonic reactions. StatPearls.

[4] Spina E (1993). Prevalence of acute dystonic reactions associated with neuroleptic treatment with and without anticholinergic prophylaxis. Int Clin Psychopharmacol.

[5] Berger JR, Vilensky JA (2014). Encephalitis lethargica (von Economo’s encephalitis). Handb Clin Neurol..

[6] Hadler NL, Roh YA, Nissan da. Oculogyric crisis after initiation of aripiprazole: A case report of an active duty service member. Case Rep Psychiatry. 2023;1–3. 10.1155/2023/9440028 PMC9845035 36660180

[7] Das S, Agrawal A (2017). Lurasidone-induced oculogyric crisis. Indian J Psychol Med.

[8] Nebhinani N, Suthar N (2017). Oculogyric crisis with atypical antipsychotics: a case series. Indian J Psychiatry.

[9] Gardner DM (2015). Incidence of oculogyric crisis and long-term outcomes with second-generation antipsychotics in a first-episode psychosis program. J Clin Psychopharmacol.

[10] Barow E (2017). Oculogyric crises: etiology, pathophysiology and therapeutic approaches. Parkinsonism Relat Disorders.

[11] Möller HJ (2005). Risperidone: a review. Expert Opin Pharmacother.

[12] Wubeshet YS, Mohammed OS, Desse TA (2019). Prevalence and management practice of first-generation antipsychotics induced side effects among schizophrenic patients at Amanuel mental specialized hospital, central Ethiopia: cross-sectional study. BMC Psychiatry.

[13] Potkin SG (2003). Aripiprazole, an antipsychotic with a novel mechanism of action, and risperidone vs placebo in patients with schizophrenia and schizoaffective disorder. Arch Gen Psychiatry.

[14] Lako IM, van den Heuvel ER, Knegtering H (2013). Estimating dopamine D 2 receptor occupancy for doses of 8 antipsychotics: a meta-analysis [J]. J Clin Psychopharmacol.

[15] de Villa AR (2022). Oculogyric crisis in the setting of low dose risperidone and benztropine mesylate use in a patient with schizophrenia: a case report and review of literature. Cureus.

[16] Masliyah T, Ad-Dab’bagh Y (2011). Low-dose risperidone-induced oculogyric crises in an adolescent male with autism, tourette's and developmental delay. J Can Acad Child Adolesc Psychiatry.

[17] Huang J, Weng S, Xie B (2000). A controlled study of clozapine in relation to delayed dyskinesia. Chinese J Neurous Ment Dis.

[18] Mehta VS, Das B (2015). Oculogyric crisis – an acute dystonia with olanzapine. J Psychiatry.

[19] Farde L, Nordström AL, Wiesel FA (1992). Positron emission tomographic analysis of central D1 and D2 dopamine receptor occupancy in patients treated with classical neuroleptics andclozapine Relation to extrapyramidal side effects. Arch Gen Psychiatry.

[20] Hongjiang D, Lan Z (2022). Oculogyric crisis caused by clozapine: a case report. Sichuan Mental Health.

[21] Schoretsanitis G, Kuzin M, Kane JM (2021). Elevated clozapine concentrations in clozapine-treated patients with hypersalivation. Clin Pharmacokinet.

